# Multispecies Outbreak of *Nocardia* Infections in Heart Transplant Recipients and Association with Climate Conditions, Australia

**DOI:** 10.3201/eid2811.220262

**Published:** 2022-11

**Authors:** Jonathan Li, Cindy Lau, Naomi Anderson, Fay Burrows, Feras Mirdad, Lilibeth Carlos, Andrew J. Pitman, Kavitha Muthiah, David R. Darley, David Andresen, Peter Macdonald, Deborah Marriott, Nila J. Dharan

**Affiliations:** St Vincent's Hospital Sydney, Darlinghurst, New South Wales, Australia (J. Li, C. Lau, N. Anderson, F. Burrows, F. Mirdad, L. Carlos, K. Muthiah, D.R. Darley, D. Andresen, P. Macdonald, D. Marriott, N.J. Dharan);; Australian Research Council Centre of Excellence for Climate Extremes and Climate Change Research Centre, at UNSW Sydney, Sydney, New South Wales(A.J. Pitman);; Victor Chang Cardiac Research Institute, Darlinghurst (K. Muthiah, P. Macdonald);; UNSW Sydney, Sydney (D.R. Darley, N.J. Dharan);; University of Notre Dame, Sydney (D. Andresen)

**Keywords:** *Nocardia*, immunocompromised host, organ transplantation, disease outbreak, climate, Australia, bacteria

## Abstract

A multispecies outbreak of *Nocardia* occurred among heart transplant recipients (HTR), but not lung transplant recipients (LTR), in Sydney, New South Wales, Australia, during 2018–2019. We performed a retrospective review of 23 HTR and LTR who had *Nocardia* spp. infections during June 2015–March 2021, compared risk factors for *Nocardia* infection, and evaluated climate conditions before, during, and after the period of the 2018–2019 outbreak. Compared with LTR, HTR had a shorter median time from transplant to *Nocardia* diagnosis, higher prevalence of diabetes, greater use of induction immunosuppression with basiliximab, and increased rates of cellular rejection before *Nocardia* diagnosis. During the outbreak, Sydney experienced the lowest monthly precipitation and driest surface levels compared with time periods directly before and after the outbreak. Increased immunosuppression of HTR compared with LTR, coupled with extreme weather conditions during 2018–2019, may explain this outbreak of *Nocardia* infections in HTR.

## Abstract 2

Extreme weather conditions, coupled with greater susceptibility to opportunistic infection, could explain this outbreak.

*Nocardia* is an environmental aerobic actinobacterium (*Actinomycete*) that stains positive on Gram stain and forms commonly in soil and water. Infection is primarily acquired through inhalation; however, it may also occur through direct inoculation into the skin or via ingestion of the microorganism (*1*,*2*). Depending on the route of infection, clinical manifestations may include pulmonary, cutaneous, intravenous line infections, and disseminated disease, which frequently involves the nervous system and skeletal or soft-tissue structures (*1*,*2*). Noncutaneous disease is most commonly reported in immunocompromised persons such as solid organ transplant recipients; recent studies showed the greatest risk is among lung transplant recipients (LTR, 3.5%) followed by heart transplant recipients (HTR, 2.5%) (*3*). Treatment in immunocompromised patients is generally for a minimum period of 6 months. Nocardiosis in solid organ transplant recipients is associated with a 10-fold increase in 1-year mortality rate (16.2%, compared with 1.3% in recipients without nocardiosis) (*4*).

In January 2018, an increased rate of *Nocardia* infections was noted among HTR at St Vincent’s Hospital in Sydney, New South Wales (NSW), Australia, but not among LTR who underwent transplants during the same timeframe. The rise in *Nocardia* infections coincided with a period of extreme weather conditions in NSW; 2018 was the second warmest and seventh driest year, and 2019 was the warmest and driest year on record in NSW (*5*,*6*). Similar extreme weather patterns were experienced across the rest of Australia (*7*,*8*). Previous studies have observed that *Nocardia* infections occur more frequently in dry and windy climates, such as that of the Southwest region of the United States (*1*,*9*). Such climate conditions are thought to increase aerosolization of *Nocardia* organisms from soil, increasing the possibility of inhalation and therefore subsequent infection (*1*,*9*,*10*).

We report an outbreak of *Nocardia* infections in HTR at St Vincent’s Hospital during January 2018–August 2019. Because *Nocardia* infections in LTR did not increase during that period, we sought to compare patient demographic characteristics, host risk factors (underlying medical conditions, rejection rates, immunosuppressive regimens), and antimicrobial prophylaxis regimens for HTR and LTR. In addition, because *Nocardia* is an environmental organism and the outbreak occurred during some of the driest years recorded in Australia, we sought to characterize climate characteristics during the time of the outbreak (*7*,*8*). St Vincent’s Hospital Human Research Ethics Committee reviewed and approved the study.

## Methods

### Study Population and Clinical Data

We conducted a retrospective review of *Nocardia* infections among HTR and LTR at St Vincent’s Hospital, Sydney. We defined a *Nocardia* case as a microbiological diagnosis of *Nocardia* in an HTR or LTR during June 2015–March 2021. Data before June 2015 were unavailable for extraction. We used the center point of a case-patient’s residential suburb as a proxy for case location to define a geographic cluster of cases as those located <5 km from the center of each cluster. We extracted clinical data from the patients’ medical records for the date of transplantation, date of *Nocardia* diagnosis, *Nocardia* species identified, site of infection, antimicrobial prophylaxis, diagnosis of other respiratory infections or cytomegalovirus (CMV) in the previous 6 months, intravenous immunoglobulin therapy, induction and maintenance immunosuppression regimens, donor and recipient CMV status, and organ rejection rates.

We defined CMV mismatch as a heart or lung transplant from a CMV-positive donor to a CMV-negative recipient. We defined CMV viremia as detectable CMV DNA (>34.5 IU/mL by Roche cobas 6800 [https://www.roche.com] CMV DNA quantitative PCR) in the 6 months preceding the *Nocardia* diagnosis. We defined significant CMV viremia as having a highest CMV DNA PCR value in the 6 months preceding *Nocardia* diagnosis >1,000 IU/mL. We defined respiratory infections by microbiological detection of pathogen using a multiplex PCR EasyScreen respiratory assay (Genetic Signatures, https://geneticsignatures.com) targeting 12 pathogens: influenza A and B, respiratory syncytial virus, parainfluenza, adenovirus, rhinovirus, metapneumovirus, seasonal coronaviruses, enterovirus, *Pneumocystis jirovecii*, *Mycoplasma pneumoniae*, and *Bordetella pertussis.*

We defined organ rejection as grade 1R or greater on endomyocardial biopsy for HTR and grade A1 or greater on transbronchial biopsy for LTR, in accordance with the International Society of Heart and Lung Transplantation 2004 and 2007 grading guidelines (*11*,*12*). At St Vincent’s Hospital, routine surveillance biopsies are performed for HTR at weeks 1, 2, 3, 4, 6, 8, and 10 and months 4, 5, 6, 8, and 10 posttransplant, and at any other time for clinically suspected rejection. In LTR, routine surveillance biopsies are performed at weeks 3, 6, and 12 posttransplant (and at week 9 if there is evidence of rejection at week 6 for early follow-up), and at any other time for substantial declines in forced expiratory volume in 1 second or for any clinically suspected rejection. In general, HTR and LTR are managed for life at our institution for any serious posttransplant complications such as opportunistic infections.

### Microbiology

We isolated *Nocardia* from induced sputum, bronchoalveolar lavage, and tissue biopsies by routine culture-based methods, including inoculation onto nonselective and enriched agar media such as horse blood, chocolate blood, and buffered charcoal yeast extract agar and incubated at 35°C. Preliminary identification was based on colony morphology, Gram stain appearance and a positive modified acid-fast stain. Mass spectrometry confirmed the identification of *Nocardia* to the genus level. Species-level identification was performed by the Institute for Clinical Pathology and Medical Research at Westmead Hospital (Sydney, NSW, Australia) and was determined by PCR and DNA sequencing of partial 5′ 16s rRNA and *Nocardia secA1* gene.

We determined in vitro antibiotic susceptibility testing by broth microdilution using a Thermo Scientific sensititer RAPMYCO AST panel (TREK Diagnostic Systems Ltd., https://www.trekds.co), performed at the reference laboratory according to manufacturer’s instructions. The antimicrobial agents tested were amoxicillin/clavulanic acid, amikacin, cefepime, cefoxitin, ceftriaxone, ciprofloxacin, clarithromycin, sulphamethoxazole, doxycycline, imipenem, linezolid, minocycline, tigecycline, and tobramycin.

### Climate Data Sources

We obtained meteorological data of monthly precipitation (mm/mo), temperature (°C), windspeed (m/sec), and evaporation (mm/mo) over the period June 2015–March 2021 from the European Centre for Medium-Range Weather Forecasts reanalysis (ERA5) (*13*) for each suburb in which *Nocardia* patients resided. ERA5 is based on the Integrated Forecasting System Cy41r2, a global numerical weather prediction system. The ERA5 reanalyses provide a gridded set of consistent daily meteorological data from 1950 through the present at 31 km spatial resolution (*13*).

We defined dryness as the ratio of actual evaporation to potential evaporation, reflecting the moisture content of the surface, such that values near 0 indicate a very dry surface, whereas values near 1.0 indicate a saturated surface. We derived erodibility as (1 – dryness) × windspeed and calculated normalized erodibility by dividing each suburb’s erodibility by the highest erodibility value experienced across all suburbs. We normalized erodibility and windspeed to transfer the spatial patterns to a 0.0–1.0 scale, which enabled us to differentiate relatively erodible locations (dry and windy) from those where erosion is likely to be low (wet and still). We chose a central location in Greater Sydney, the suburb of Greystanes, as the reference site to compare meteorological reanalysis data for all *Nocardia* infections in Greater Sydney. The benchmark for Australia and NSW comparisons was averaged values during 1961–1990 because this period includes the satellite record that is important for the reanalyses and is used as the global standard period for comparison globally (*13*).

### Statistical Analyses 

We performed Mann-Whitney U test to compare the median age at *Nocardia* diagnosis and the median time from transplant to diagnosis in HTR and LTR. We performed a z-score test for 2 population proportions to compare demographic, clinical, and microbiologic characteristics between HTR and LTR. We used a 1-way analysis of variance test to compare differences in the meteorological reanalysis data in Greater Sydney in the periods of June 2015–December 2017 (before the outbreak), January 2018–December 2019 (during the outbreak) and January 2020–March 2021 (after the outbreak). We considered a p value <0.05 statistically significant. We analyzed all data using Microsoft Excel version 16.53 with Analysis ToolPak (Microsoft, https://www.microsoft.com). 

## Results

**Figure 1:**
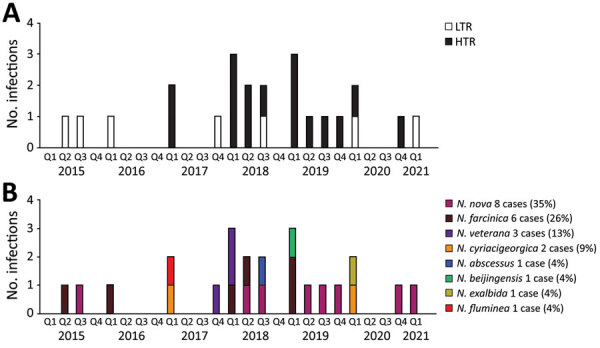
*Nocardia* infections among HTR and LTR, by date of first positive specimen, Greater Sydney, New South Wales, Australia, June 2015–March 2021. During June 2015–December 2017, there were 6 *Nocardia* cases (0.19/mo), of which 2 (33%) were in HTR. During January 2018–December 2019, there were 13 *Nocardia* case-patients (0.54/mo), of which 12 (92%) were in HTR. During January 2020–March 2021, there were 4 *Nocardia* cases (0.26 per month) of which 2 (50%) were HTR. A) *Nocardia* cases over time by type of transplant. B) *Nocardia* cases over time by *Nocardia* species. HTR, heart transplant recipient; LTR, lung transplant recipient.

We identified a total of 23 cases of *Nocardia* in heart and lung transplant recipients at St Vincent’s Hospital during June 2015–March 2021; of those, 16 were HTR and 7 were LTR (Figure 1, panel A). Compared with the years before and after the outbreak period (2018–2019), we saw no significant change in the annual total of heart and lung transplants performed (40–50/year). Over the entire study period, the total number of transplants performed was similar across the 2 services: 289 lung transplants and 275 heart transplants. There was no increase in the number of cases of *Nocardia* across other hospital departments during the study period: 2 cases during the preoutbreak period (June 2015–December 2017), 3 during the outbreak period (January 2018–December 2019), and 2 during the postoutbreak period (January 2020–March 2021).

Overall, the median age at *Nocardia* diagnosis was 59 (range 38–71) years; 15 (65.2%) patients were male (Table 1). Sites of *Nocardia* infection were lung (11 HTR, 4 LTR); bloodstream (1 HTR); skin (1 HTR); lung and bloodstream (1 LTR); lung and brain (2 HTR); skin and brain (1 LTR); lung, skin, and brain (1 LTR); and lung, brain, and kidney (1 HTR).

**Table 1:** Clinical characteristics of heart and lung transplant recipients with confirmed *Nocardia* infection, Greater Sydney, New South Wales, Australia, June 2015–March 2021*

Characteristic	Heart transplant, n = 16	Lung transplant, n = 7	p value	All patients, n = 23
Median age at *Nocardia* diagnosis, y (range)	61 (38–71)	59 (50–70)	0.98	61 (38–71)
Sex				
M	10 (62.5)	5 (71.4)	0.68	15 (65.2)
F	6 (37.5)	2 (28.6)	0.68	8 (34.8)
Median no. months from transplant to *Nocardia* diagnosis (range)	4.8 (3–19)	22.8 (5–263)	<0.01	6.3 (3–263)
CMV, donor positive/recipient negative	4 (25.0)	0	0.17	4 (17.4)
Episodes of organ rejection from date of transplant to diagnosis of *Nocardia*				
Any grade†	16 (100)	3 (42.9)	<0.01	19 (82.6)
<2R/A3	6 (37.5)	3 (42.9)	0.81	9 (39.1)
>2R/A3	10 (62.5)	0	<0.01	10 (43.4)
Diabetic at time of diagnosis	13 (81.3)	2 (28.6)	<0.01	15 (65.2)
Received intravenous immunoglobulin therapy	7 (43.8)	6 (85.7)	0.06	13 (56.5)
Respiratory virus <6 mo before *Nocardia*	9 (56.3)	6 (85.7)	0.17	15 (65.2)
CMV DNA detected by PCR <6 mo before *Nocardia*	6 (37.5)‡	1 (14.3)§	0.27	7 (30.4)
Significant CMV viremia <6 mo before *Nocardia¶*	4 (25.0)	1 (14.3)	0.57	5 (21.7)
Medications received				
Sulfamethoxazole/trimethoprim prophylaxis	13 (81.3)	7 (100)	0.22	20 (87.0)
Azithromycin prophylaxis	1 (6.3)	7 (100)	<0.01	8 (34.8)
Induction with basiliximab	16 (100)	2/5 (40.0)	<0.01	18/21 (85.7)
Tacrolimus immunosuppression	16 (100)	7 (100)	1	23 (100)
Mycophenolic acid immunosuppression	16 (100)	5/5 (100)	1	21/21 (100)
Prednisone immunosuppression	16 (100)	5/5 (100)	1	21/21 (100)

*Values are no. (%) patients except as indicated. Denominators are indicated for categories in which only some patients had data available. CMV, cytomegalovirus. †Defined as >1R on endomyocardial biopsy for heart transplant rejections and >A1 on bronchial biopsy for lung transplant rejections in accordance with International Society of Heart and Lung Transplantation 2004 and 2007 grading guidelines. ‡Among heart transplant recipients, the highest CMV PCR values (IU/mL) included: 1,169 copies 3 mo before *Nocardia* diagnosis; 16,271 4 mo before *Nocardia* diagnosis; 4,446 3 mo before *Nocardia* diagnosis; 27,1942 within a month before *Nocardia* diagnosis; 324 within a month before *Nocardia* diagnosis; and 103 within 1 month before *Nocardia* diagnosis. §The highest CMV PCR value (IU/mL) was 1,240 within a month before *Nocardia* diagnosis. ¶Defined as CMV PCR copies >1,000 IU/mL.

When comparing heart and lung transplant recipients, we saw no substantial differences in age at *Nocardia* diagnosis, sex, CMV mismatch status, use of intravenous immunoglobulin, CMV viremia, significant CMV viremia, diagnosis of other respiratory infections within 6 months preceding the *Nocardia* diagnosis, or use of sulfamethoxazole/trimethoprim for *Pneumocystis jirovecii* prophylaxis. In addition, all heart and lung transplant recipients received a similar combination of maintenance immunosuppression therapy with tacrolimus, mycophenolate mofetil, and prednisone.

We identified significant differences between LTR and HTR that suggested greater immunosuppression in HTR before and at the time of *Nocardia* diagnosis (Table 1). These differences included a shorter median time from transplant to *Nocardia* diagnosis (4.8 months, vs. 22.8 months in LTR; p<0.05), higher prevalence of diabetes at the time of *Nocardia* diagnosis (81.3%, vs. 28.6% in LTR; p<0.05), greater use of basiliximab for induction immunosuppression (100%, vs. 40.0% in LTR; p<0.05), increased rates of any grade of cellular rejection at any time before *Nocardia* diagnosis (100%, vs. 42.9% in LTR; p<0.05), and increased rates of moderate to severe rejection at any time before *Nocardia* diagnosis (62.5%, vs. 0 in LTR; p<0.05). In addition, azithromycin prophylaxis rates were lower in HTR (6.25%) than in LTR (100%) (p<0.05).

We found no significant difference in the types of *Nocardia* species between HTR and LTR (Table 2) and no apparent clustering of *Nocardia* species over time to suggest a single source of the outbreak (Figure 1, panel B). We also found no differences between HTR and LTR in *Nocardia* susceptibilities to amikacin, amoxicillin/clavulanic acid, ceftriaxone, ciprofloxacin, clarithromycin, doxycycline, imipenem, linezolid, minocycline, moxifloxacin, and tobramycin (Table 3). *Nocardia* in LTR were more likely to be susceptible to cefepime than in HTR (50% vs. 6.7%; p<0.05), however, this difference may have been related to differences in *Nocardia* species across type of transplant.

**Table 2:** Comparison of *Nocardia* species infecting heart and lung transplant recipients, Greater Sydney, New South Wales, Australia, June 2015–March 2021*

*Nocardia* species	Heart transplant, n = 16	Lung transplant, n = 7	p value	All patients, n = 23
*N. abscessus *	0	1 (14)	0.12	1 (4)
*N. beijingensis*	1 (6)	0	0.50	1 (4)
*N. cyriacigeorgica*	2 (13)	0	0.33	2 (9)
*N. exalbida*	0	1 (14)	0.12	1 (4)
*N. farcinica*	4 (25)	2 (29)	0.85	6 (26)
*N. fluminea*	1 (6)	0	0.50	1 (4)
*N. nova*	6 (38)	2 (29)	0.68	8 (35)
*N. veterana*	2 (13)	1 (14)	0.91	3 (13)

*Values are no. (%) patients except as indicated.

**Table 3:** Comparison of the number and proportion of *Nocardia* isolates susceptible to select antimicrobial drugs between heart and lung transplant recipients, Greater Sydney, New South Wales, Australia, June 2015–March 2021*

Drug	Heart transplant, n = 16	Lung transplant, n = 7	p value	All patients, n = 23
Amikacin	16 (100)	7 (100)	Referent	23 (100)
Augmentin	0	0/6	Referent	0/22 (0)
Cefepime	1/15 (6.7)	3/6 (50.0)	0.02	4/21 (19.0)
Ceftriaxone	3 (18.8)	4 (57.1)	0.07	7 (30.4)
Ciprofloxacin	4 (25.0)	1 (14.3)	0.57	5 (21.7)
Clarithromycin	10 (62.5)	4 (57.1)	0.81	14 (60.9)
Doxycycline	1/15 (6.7)	2 (28.6)	0.16	3/22 (13.6)
Imipenem	7 (43.8)	3 (42.9)	0.97	10 (43.5)
Linezolid	16 (100)	7 (100)	Referent	23 (100)
Minocycline	1 (6.3)	2 (28.6)	0.14	3 (13.0)
Moxifloxacin	4 (25.0)	1/6 (16.7)	0.68	5/22 (22.7)
Tobramycin	4 (25.0)	2/6 (33.3)	0.70	6/22 (27.3)]
Sulfamethoxazole/trimethoprim	15 (93.8)	7 (100)	0.50	22 (95.7)

*Values are no. (%) patients except as indicated. Denominators are indicated for categories in which only some patients had data available.

**Figure 2:**
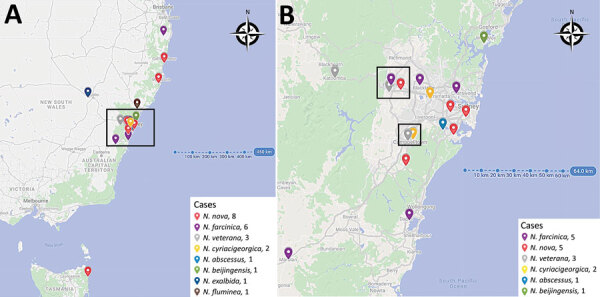
Location of *Nocardia* infections among heart and lung transplant recipients identified at St Vincent’s Hospital, Sydney, New South Wales, Australia, June 2015–March 2021. A) All *Nocardia* cases. Box indicates Greater Sydney region. B) All *Nocardia* cases within the Greater Sydney region.

Six *Nocardia* cases were located outside Greater Sydney, and 17 were within the Greater Sydney region (Figure 2). During June 2015–March 2021, no *Nocardia* patients changed their residential address within 3 months before *Nocardia* diagnosis. The 6 *Nocardia* cases outside Greater Sydney were situated in northern coastal NSW (n = 4), central NSW (n = 1), and Tasmania (n = 1). In western Sydney, 2 clusters of *Nocardia* were in geographic proximity (Figure 2, panel B). However, those clusters did not represent an outbreak because different species were identified within each cluster and cases diagnosed in the clusters were separated in time by >6 months.

**Figure 3:**
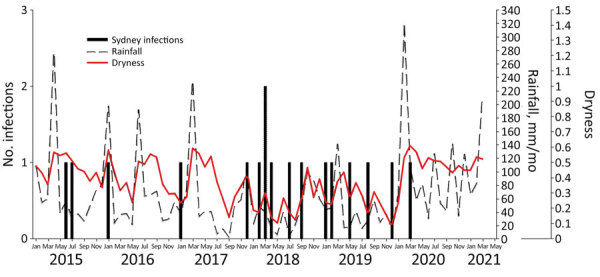
*Nocardia* cases among heart and lung transplant recipients compared with precipitation and dryness in Greystanes, the central location for cases in Greater Sydney, New South Wales, Australia, June 2015–March 2021. We defined dryness as the ratio of evaporation to potential evaporation, such that 0.0 is perfectly dry and 1.0 is perfectly wet.

Because 74% of cases were from Greater Sydney, our climate data analysis focused on that region. We chose the suburb of Greystanes as the reference location for all climate data comparisons because of its central geographic location. Five *Nocardia* cases were reported from Greater Sydney during June 2015–December 2017 (0.16/month), of which 1 (20%) was an HTR. During January 2018–December 2019, a total of 11 *Nocardia* cases (0.46/month) were diagnosed, of which 10 (91%) were HTR; during January 2020–March 2021, there was 1 *Nocardia* case (0.07/month) in an HTR (Figure 3). The increased incidence of *Nocardia* from January 2018 to December 2019 occurred during times of lower rainfall and a drier surface (Figure 3).

When comparing averaged values of climate parameters in Greater Sydney between the time of the outbreak (January 2018–December 2019), and the periods before (June 2015–December 2017) and after (January 2020–March 2021), we found the outbreak coincided with average monthly precipitation of 40.0 mm/month, significantly lower than that for the time periods before and after (p<0.01) (Table 4). During the same period, average monthly dryness reached its lowest (driest) value of 0.3 compared with the time periods before and after (p<0.01). We observed no significant differences in the average monthly temperature, windspeed, or erodibility during the outbreak period (January 2018–December 2019) (Table 4), compared with the time periods before and after. However, when we compared months with a *Nocardia* diagnosis with months without a *Nocardia* diagnosis, windspeed was higher (0.4 vs. 0.2 m/s; p<0.01) and surface levels were drier (0.3 vs. 0.4; p<0.01); because erodibility is derived from dryness and windspeed, erodibility was also higher (0.4 vs. 0.1 m/s for months without a *Nocardia* diagnosis; p<0.01). The average temperature was also higher in months with a *Nocardia* diagnosis (18.9°C vs. 17.4°C in months without a *Nocardia* diagnosis), although this difference was not statistically significant (p<0.24) (Table 5).

**Table 4:** Climate conditions and *Nocardia* incidence before, during, and after a *Nocardia* outbreak among heart and lung transplant recipients, Greater Sydney, New South Wales, Australia, January 2018–December 2019*

Characteristic	Preoutbreak, Jun 2015–Dec 2017	Outbreak, Jan 2018–Dec 2019	Postoutbreak Jan 2020–Mar 2021	p value
*Nocardia* cases/mo	0.16	0.46	0.07	
Average monthly precipitation, mm/mo	60.4	40.0	103.2	<0.01
Average monthly temperature °C	17.0	17.8	17.8	0.79
Average monthly dryness*	0.4	0.3	0.5	<0.01
Average monthly windspeed, m/s	1.0	0.9	1.0	0.62
Average monthly erodibility†	0.1	0.2	0.1	0.26

*Ratio of evaporation to potential evaporation, such that 0.0 is perfectly dry and 1.0 is perfectly wet. †Calculated by the formula (1 – dryness) × windspeed.

**Table 5:** Comparison of climate conditions during months with and without *Nocardia* infections, Greater Sydney, New South Wales, Australia, June 2015–March 2021

Climate conditions	Monthly average with no *Nocardia* cases, n = 73	Monthly average with confirmed *Nocardia* cases, n = 17	p value	All months, n = 90
Precipitation, mm/mo	66.3	63.3	0.42	65.7
Dryness	0.4	0.3	<0.01	0.4
Temperature, °C	17.4	18.9	0.24	17.7
Windspeed, m/s	1.0	1.1	0.37	1.0
Normalized windspeed, m/s	0.2	0.4	<0.01	0.2
Normalized erodibility, m/s	0.1	0.4	<0.01	0.2

## Discussion

In this study, we observed no differences in *Nocardia* species isolated from HTR compared with LTR and no clustering of *Nocardia* species in space or time to suggest a single source for the outbreak. We found that HTR had evidence of greater immunosuppression before their *Nocardia* diagnosis than LTR, including higher use of basiliximab for induction immunosuppression, higher rates of cellular rejection, and a shorter median time from transplant to *Nocardia* diagnosis. Our analysis of climate data revealed that low local precipitation and drier surface levels correlated with increased incidence of *Nocardia* diagnosis.

Previous studies have shown that high-dose steroids, calcineurin inhibitor usage, CMV disease in the 6 months before diagnosis, and increased age are risk factors for nocardiosis (*3*,*14*). In our cohort, HTR and LTR did not have demonstrable differences in age, CMV disease status, or use of tacrolimus, mycophenolate mofetil, or prednisone. However, our data indicate that HTR had more characteristics indicating immunosuppression than LTR, including higher rates of basiliximab induction, diabetes, and cellular rejection (including treated moderate-to-severe rejection). Because basiliximab is an immunosuppressant with interleukin-2R α antagonist properties, increased use of basiliximab among HTR compared with LTR may have increased the risk for *Nocardia* infection through a diminished T-cell response in HTR. Similarly, additional immunosuppression in the setting of treating moderate to severe acute organ rejection may have further increased the risk for *Nocardia* infection among HTR.

We found no difference in the proportion of macrolide susceptible *Nocardia* isolates in HTR versus LTR despite increased use of azithromycin for prophylaxis in LTR compared with HTR. The possibility that azithromycin use lowered the risk for *Nocardia* infections in LTR compared with HTR warrants further investigation.

Seasonality and changes in climate conditions are known to affect the incidence and distribution of various infectious pathogens, particularly those acquired by inhalation or exposure to the respiratory tract (*15*). Respiratory viruses and environmental fungi, such as *Coccidioides immitis*, *Aspergillus* spp., and *Cryptococcus gattii*, are highly influenced by climate patterns, having enhanced infection risk in the setting of increased aerosolised dust particulate matter (*9*,*10*,*16*,*17*). In a study of *Aspergillus* infections among stem cell transplant recipients in the United States, Panackal et al. identified an increased incidence of invasive *Aspergillosis* in drier and warmer conditions by comparing incidence rates across different seasons (*16*). Tong et al. identified a correlation between increased frequencies of dust storms, precipitated by low moisture level soils, and higher rates of inhaled soil-dwelling fungi such as *Coccidioides immitis* and *C. posadasii* (*17*). Similarly, Majeed et al. (*9*) and Saubolle et al. (*10*) observed that the greatest number of *Nocardia* infections occur in dry, warm climates, such as in the Southwest United States.

In Greater Sydney, the increase in *Nocardia* cases occurred during a time of decreased rainfall and a very dry surface (evaporation/potential evaporation), supporting our hypothesis that the increase in *Nocardia* infections may have been driven by extreme climate conditions. In NSW, 2019 was the warmest and driest year, with the annual mean temperature 1.95 degrees above average and average rainfall 55% below average (*6*); 2018 was NSW’s second warmest and seventh driest year, with an annual mean temperature 1.68 degrees above average and average rainfall 40% below average (*5*).

The Australian Therapeutic Guidelines for treating moderate nocardiosis recommend dual therapy with sulfamethoxazole/trimethoprim and either ceftriaxone or linezolid; for severe nocardiosis, a third agent (amikacin, imipenem or meropenem), is added to this regimen (*18*). Most *Nocardia* isolates identified in this cohort were reassuringly susceptible to sulfamethoxazole/trimethoprim (95.7%); however, only 30.4% of isolates were susceptible to ceftriaxone and 43.5% to imipenem, corresponding well with previous data (*19*). All isolates were susceptible to linezolid and amikacin, supporting the usage of empiric linezolid or amikacin in addition to sulfamethoxazole/trimethoprim over ceftriaxone or imipenem for moderate and severe disease in our cohort.

Our study’s first limitation is that our small study population size limited our analyses to primarily descriptive statistics and correlations with climate data. Most of our patients were from the Greater Sydney region and underwent heart or lung transplantation at a single medical center, further limiting the generalizability of our findings. The retrospective study design and reliance on data from the medical record limited our ability to comment on patient practices, including use of personal protective equipment such as masks when gardening and participating in other activities that may increase the risk for infection. However, postoperative patient education on use of personal protective equipment did not change during the study period; we would therefore not expect to see changes in patient practices that would affect the incidence of *Nocardia* infections. The retrospective study design meant that data on intraoperative care, such as changes in procedures, personnel, and infection control practices, were not available. However, because the surgical teams and theater conditions remained consistent throughout the study period, differences in intraoperative care that may have affected risk for *Nocardia* infection would be unlikely. Data on environmental sources within and around the hospital, such as construction projects and water sources, were also not available. However, given the broad range in times from transplant to diagnosis and the multispecies nature of the outbreak, it is unlikely that environmental contamination was a potential source of the outbreak. Although more surveillance biopsies are routinely performed among HTR than LTR at our institution, which may have contributed to the higher rates of any grade of rejection detected in HTR, treated rejection episodes (i.e., moderate to severe rejection) are likely to be symptomatic and therefore unlikely to be biased by differences in routine surveillance biopsy schedules. *Nocardia* infections can be indolent and subclinical for some time, which limited our analyses comparing climate conditions in months with and without *Nocardia* diagnoses. Larger studies and studies in other regions are needed to confirm the correlations we identified between climate conditions and *Nocardia* infections.

In conclusion, tThis study examined the association between climate conditions and *Nocardia* infection in HTR and LTR. Periods of low precipitation and dryer surface levels were associated with an increased incidence of *Nocardia* diagnoses, suggesting that environmental conditions affected the risk for *Nocardia* infection. In addition, HTR had a shorter time to *Nocardia* diagnosis and increased rates of markers of immunosuppression, suggesting that HTR had greater susceptibility to infection at the time of diagnosis than LTR. We hypothesize that the increased environmental risk from climate conditions during 2018–2019, coupled with increased host susceptibility related to immunosuppression in HTR, may explain the increased incidence of *Nocardia* infections during 2018–2019. Further studies should evaluate the influence of climate characteristics on *Nocardia* infections in immunocompromised hosts, as well as potential screening or other preventive measures that might decrease disease burden in these vulnerable patients. Because *Nocardia* is an environmental organism, these results highlight the importance of wearing personal protective equipment around soil exposures, particularly for immunocompromised patients during periods of increased soil dryness.
